# Correlation of Aurora-A expression with the effect of chemoradiation therapy on esophageal squamous cell carcinoma

**DOI:** 10.1186/s12885-015-1329-3

**Published:** 2015-04-29

**Authors:** Kiyokazu Tamotsu, Hiroshi Okumura, Yasuto Uchikado, Yoshiaki Kita, Ken Sasaki, Itaru Omoto, Tetsuhiro Owaki, Takaaki Arigami, Yoshikazu Uenosono, Akihiro Nakajo, Yuko Kijima, Sumiya Ishigami, Shoji Natsugoe

**Affiliations:** Department of Digestive Surgery, Breast and Thyroid Surgery, Graduate School of Medical Sciences, Kagoshima University, Sakuragaoka 8-35-1, Kagoshima, 890-8520 Japan

**Keywords:** Aurora-A, Chemoradiation, Esophageal cancer

## Abstract

**Background:**

Chemoradiation therapy (CRT) is one of the most useful treatments for esophageal squamous cell carcinoma (ESCC). However, because some patients respond well to CRT and others do not, it is important to be able to predict response to CRT before beginning treatment by using markers. Aurora-A encodes a cell cycle regulated serine/threonine kinase that has essential functions in centrosome maturation and chromosome segregation. In this study, we investigated the relationship between the expression of Aurora-A and the response to CRT in patients with ESCC.

**Methods:**

We immunohistochemically investigated the expression of Aurora-A in biopsy specimens of untreated primary tumors of 78 patients with ESCC and determined the relationship between Aurora-A levels and patient responses to CRT, which consisted of 5-fluorouracil plus cisplatin and 40 Gy of radiation.

**Results:**

Tumors were judged as Aurora-A positive when more than 10% of the cancer cells displayed a distinct positive nuclear anti-Aurora-A immunoreaction by immunohistochemical evaluation. The tumors of 46 of 78 patients (58.9%) displayed positive expression of Aurora-A. In terms of clinical response the percentage of patients showing complete response (CR), incomplete response/stable disease of primary lesion (IR/SD), and progressive disease (PD) was 19.2, 69.2, and 11.5%, respectively. In terms of histological response the tumor grade of the 41 patients who underwent surgery was as follows: grade 1, 48.8%; grade 2, 29.2%; grade 3, 22.0%. CRT was effective for patients who had Aurora-A (+) tumors (clinically: P = 0.0003, histologically: P = 0.036).

**Conclusions:**

Our results suggest that Aurora-A expression in biopsy specimens of primary tumors is associated with CRT efficacy in patients with ESCC. Assessment of Aurora-A expression in biopsy specimens maybe useful for regarding the potential utility of CRT therapy for patients with ESCC before treatment.

## Background

Esophageal cancer is one of the most malignant cancers with an extremely poor prognosis even though various types of aggressive therapy such as extended lymphadenectomy, radiotherapy, chemotherapy and chemoradiation therapy (CRT) have been used [1-4]. Recent fundamental research indicated that many biological markers associated with apoptosis, DNA repair and the cell cycle such as tumor suppressors (p53, p21), cell-cycle regulators (cyclin D1, CDC25B, 14-3-3 sigma), DNA repair (p53R2, ERCC1) are associated with response to CRT in esophageal squamous cell carcinoma (ESCC) [5-7]. Most cancers lack regulation of the cell cycle and cell cycle checkpoints, resulting in diseases of uncontrolled proliferation [8]. Of the molecules that are associated with cell cycle checkpoints and mitosis, Aurora kinase is a key protein that plays a role in cell proliferation. Aurora-A has been characterized as a mitotic kinase and encodes a cell cycle regulated serine/threonine kinase that has essential functions in centrosome maturation and chromosome segregation. Aurora kinase activity regulates the G2 to M phase transition of the cell cycle [9]. Recently, overexpression of Aurora-A was detected in a variety of human cancers such as carcinomas of the breast, esophagus, pancreas, liver, bladder and ovary [9-15]. The purpose of this study was to examine the correlation of Aurora-A expression with the effect of CRT in ESCC.

## Methods

This study was approved by the ethics committee of Kagoshima University.

### Study groups and patient characteristics

The present study group involved 78 consecutive patients with advanced ESCC who underwent CRT at Kagoshima University Hospital between 1995 and 2006. Of these patients, 41 patients underwent CRT followed by esophagectomy with lymph node dissection 4–6 weeks after completing CRT, and 37 patients received only CRT. Informed consent was obtained from all patients and biopsy specimens of the primary tumors were collected by endoscopic examination before CRT. The general condition and tumor stage of the patients were evaluated before and after CRT by performance status and by imaging means such as esophagoscopy, esophagography, computed tomography and endoscopic ultrasonography. Tumor stage was based on the International Union Against Cancer tumor-node-metastasis (TNM) classification system [16]. In this period, most of patients were treated with preoperative chemotherapy and we adopted CRT to relatively advanced patients after informed consent. Moreover we did not adopt CRT to the patients with synchronous or metachronous cancer in other organs. Therefore the patient’s number was limited. However, during this period, the patients were treated by same surgical team under same treatment strategies.

The study group consisted of 2 patients with stage I, 12 patients with stage II, 30 patients with stage III and 34 patients with stage IV tumors. Thirty-seven patients were treated with only CRT, including 15 patients with tumor invasion to adjacent structures by preoperative diagnosis, 5 patients with refusal of surgery and 17 patients with poor condition because of their complications. Thus, 41 patients were judged to be eligible for curative resection after completing neoadjuvant CRT. The TNM classification of these 41 patients was as follows; 2, 0, 25 and 14 patients were cT1, T2, T3 and T4 tumors respectively, 13 and 28 cases were cN0 and N1 respectively, 28 and 13 cases were cM0 and M1 respectively and 1, 9, 15 and 16 cases were cStage I, II, III and IV, respectively. During operation, all cT4 tumors including three patients with T4 tumors invading to the lung were judged to be resectable. All of the M1 tumors were due to distant lymph node metastases. These 41 patients underwent esophagectomy with lymph node dissection and subsequently cervical esophagogastric anastomosis using a gastric tube was performed. The biopsy specimens taken were two specimens from carcinoma lesions and one specimen from normal epithelia. Additional specimens were taken when there were dysplastic lesions. All patients were followed up after discharge with a radiographic examination every 1–3 months, computed tomography every 3–6 months, and ultrasonography every 6 months. Follow-up data after surgery were available for all patients with a median follow-up period of 24 months (range 3–136 months). The clinicopathologic features of the study group are summarized in Table 1.

**Table 1:** **Patient characteristics**

Characteristics	No.
Gender (male/female)	75/3
Age (yrs)	64.5 (42–84)
Tumor location	
Upper/middle/lower	19/43/16
Histological type	
Well/mode/poor	11/44/23
cT	
T1/T2/T3/T4	5/3/41/29
cN	
N0/N1	17/61
cM	
M0/M1	60/18
cStage	
I/II/III/IV	2/12/30/34

### Chemoradiation therapy

The total radiation dose administered was 40 Gy. 2 Gy fractions were delivered to the mediastinum and neck 5 days per week for 4 weeks. During the same period, chemotherapy was administered intravenously using cisplatin (7 mg/m2) and 5 fluorouracil (350 mg/m2) (4). For the patients treated without surgery, definitive CRT (a total radiation dose was more than 50 Gy) was applied. After 4 weeks, the clinical response to CRT was evaluated based on the findings of esophagography, esophagoscopy, endoscopic ultrasonography and computed tomography. The clinical criteria for response of the primary lesion were as follows [17]: Complete response (CR); disappearance of endoscopic findings that suggest the presence of a tumor, no malignant cell by endoscopic biopsy from the area where the primary tumor had existed, the entire esophagus can be observed by endoscopy, and no findings of active esophagitis by endoscopy. Incomplete response/stable disease of primary lesion (IR/SD); response of the primary lesion is judged as IR/SD when its response does not meet the conditions for complete response or progressive disease. Progressive disease (PD); distinct tumor growth or progression in esophageal stenosis compared with the best condition during treatment.

The histological criteria for response to CRT were as follows [17]. Grade 0; neither necrosis nor cellular or structural changes can be seen throughout the lesion. Grade 1; necrosis or disappearance of the tumor is present in no more than 2/3rds of the whole lesion. Grade 2; necrosis or disappearance of the tumor is present in more than 2/3rds of the whole lesion, but viable tumor cells still remain. Grade 3; the whole lesion displays necrosis and/or is replaced by fibrosis, with or without granulomatous changes; no viable tumor cells are observed. A response of Grade 2 or 3 was judged as effective CRT and a response of Grade 0 or 1 was judged as ineffective CRT.

### Immunohistochemical examination

Aurora-A protein expression was determined using an immunohistochemical method. Tumor samples were fixed with 10% formaldehyde in phosphate-buffered saline (PBS), embedded in paraffin, and section into 4 μm-thick slices. For each tumor sample, three biopsy specimens, including two cancerous lesions and one normal epithelium were mounted alongside each other on a slide glass. Paraffin-embedded sections were dewaxed in xylene and rehydrated in a graded series of ethanol. After deparaffinization of the sections, endogenous peroxidase was blocked by immersing the slides in a 0.3% hydrogen peroxidase-methanol solution for 30 minutes at room temperature. In preparation for staining with primary antibody, the sections were pretreated with 0.1 M citrate buffer for 10 minutes at 120°C in an autoclave. The sections were then incubated with the primary antibody, anti-Aurora-A antibody (Cell Signaling Technology#3092) 1:50 diluted with Antibody Diluent (Dako, Inc) at 4°C overnight [10], followed by staining using a streptavidin-biotin-peroxidase kit (Nichirei, Tokyo, Japan). The sections were washed three times with PBS for 5 min per wash, and the immune complex was visualized by incubating the sections with diaminobenzidine tetrahydrochloride. The slides were rinsed briefly in water, counterstained with haematoxylin and mounted. Esophageal cancer sections that are known to express Aurora-A were used as positive control slides and a section without primary antibody was used as a negative control.

The sections were examined under a light microscope. Evaluation of immunohistochemical data was independently carried out by two investigators (K.T. and H.O.) without prior knowledge of patient clinical information. Positive staining of the nucleus was evaluated in all areas of the specimen. To establish the cut-off of Aurora-A expression we have tested the significance value of correlation between CRT response and Aurora-A expression according to Aurora-A expression rates, such as 10%, 20%, 30%, 40%, and more than 50%, then best significance was obtained at 10%. Therefore, tumors were classified as Aurora-A positive when >10% of the tumor nuclei were stained.

### Statistical analysis

Statistical analysis of group differences was performed using the χ2 test. A value of P < 0.05 was considered to be significant. Actuarial survival curves were estimated using the Kaplan–Meier method, and differences in survival between subgroups were compared with the log-rank test and Wilcoxon test. Multivariate analysis was performed using Cox-hazard model analysis. A p value of < 0.05 was considered to be significant. All p-values are two-sided in this study. All statistical analyses were performed using the software package StatView™ version 5.0 (Abacus Concepts, Berkeley, CA, US).

## Results

### Expression of Aurora-A in ESCC

**Figure 1:**
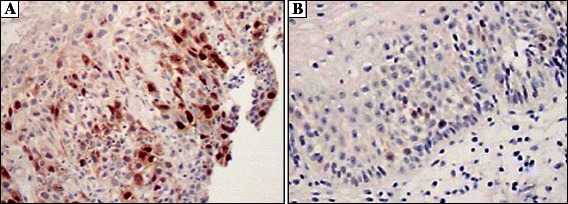
Expression of Aurora-A in clinical samples of ESCC tumors. Immunostaining of Aurora-A in representative tumors (original magnification, ×400): **(A)** Aurora-A positive ESCC; **(B)** Aurora-A negative ESCC. Positive distinct Aurora-A staining is detected in the cell nucleus.

Aurora-A expression in ESCC tumor samples was immunohistochemically detected in both the nucleus and the cytoplasm of the cells. The patterns of expression observed were distinct nuclear expression, or diffuse nuclear and cytoplasmic expression. Since the dominant cellular localization of Aurora-A is the centrosome, we counted tumors with distinct nuclear staining as Aurora-A positive (Figure 1). Of 78 ECC patients, 58.9% were positive for Aurora-A expression.

### Relationships between Aurora-A expression and response to CRT

The percentage of patients with a CR, IR/SD or PD clinical response to CRT was: 19.2% (15 out of 78), 69.2% (54 out of 78) and 11.5% (9 out of 78), respectively. Analysis of the relationship between the expression of Aurora-A and the clinical response to CRT indicated that CRT was effective in patients who had Aurora-A (+) tumors (P = 0.0003) (Table 2).

**Table 2:** **Correlation of Aurora-A expression with clinical response to CRT**

	Clinical response to CRT (n = 78)				
	CR	IR/SD	PD	Total	*P*
Aurora-A					
(+)	15	29	2	46	0.0003
( − )	0	25	7	32	

CR: Complete Response, complete disappearance of the primary lesion.

IR/SD: Incomplete Response/Stable Disease of the primary lesion.

PD: Progressive Disease, progressive disease of the primary lesion.

Aurora-A (+)/(−), Aurora-A positive/negative expression.

The grades of the histological response of the 41 patients who underwent surgery were as follows: grade 1, 48.8% (20 out of 41 patients); grade 2, 29.2% (12 out of 41 patients) and grade 3, 22.0% (9 out of 41 patients). In total, the response of 21 patients (51.2%) with grade 2 or 3 was judged as effective CRT, whereas the response of 20 patients (47.2%) with grade 1 was judged as ineffective CRT. There was a significant correlation between the pathological and clinical responses to CRT (p = 0.0001). Analysis of the correlation of Aurora-A expression with histological effect again indicated that CRT was effective for patients with Aurora-A (+) tumors (P = 0.003) (Table 3).

**Table 3:** **Correlation of Aurora-A expression with histological and clinical responses to CRT in the surgical group of ESCC patients**

	Histological response to CRT (n = 41)				
	Grade1	Grade2	Grade3	Total	*P*
Aurora-A					
(+)	7	8	9	24	0.003
( −)	13	4	0	17	
Clinical response					
CR	0	1	8	9	0.0001
IR/SD	17	11	1	29	
PD	3	0	0	3	

Grade 2 or 3 was judged as effective CRT and a response of Grade 1 was judged as ineffective CRT.

CR: Complete Response, complete disappearance of the primary lesion.

IR/SD: Incomplete Response/Stable Disease of the primary lesion.

PD: Progressive Disease, progressive disease of the primary lesion.

Aurora-A (+)/(−), Aurora-A positive/negative expression.

### Clinical outcomes according to Aurora-A expression or CRT response

**Figure 2:**
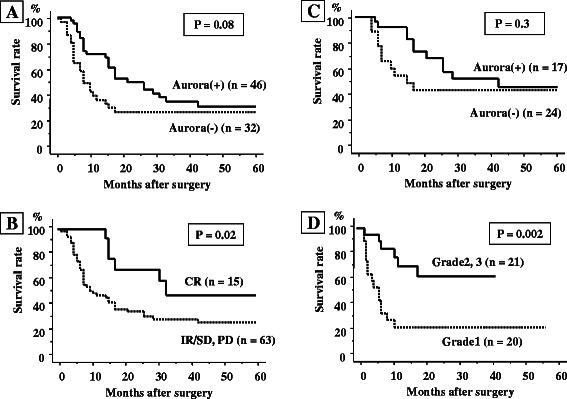
Disease-specific survival curves of ESCC patients treated with CRT and surgery according to Aurora-A expression. **(A)** Of 78 patients, the 2-year or 5-year survival rates of patients with Aurora-A (+) and Aurora-A (−) tumors were 49.0 and 28.1% or 29.5 and 28.1%, respectively (P = 0.08). **(B)** Of 78 patients, the 5-year survival rate of patients with CR and IR/SD, or with PD clinical responses to CRT were 46.0 and 27.3%, respectively (P = 0.02). **(C)** Of the 41 patients who underwent surgery, the 2-year or 5-year survival rates of patients with Aurora-A (+) and Aurora-A (−) tumors were 64.8 and 41.2 or 47.1and 40.2%, respectively (P = 0.3). **(D)** The 5-year survival rate of patients with grade 2 or 3, or with grade 1 tumors was 61.8 and 21.2%, respectively (P = 0.002). P-values were calculated using log-rank tests.

The relationship between the expression of Aurora-A and clinical outcome of the 78 patients was next analyzed. The 2-year or 5-year survival rates of patients with Aurora-A (+) and Aurora-A (−) tumors were 49.0 and 28.1% or 29.5 and 28.1%, respectively (P = 0.08, Figure 2A). Thus the patients with Aurora-A (+) tumors tended to have a better prognosis than the patients with Aurora-A (−) tumors. When analyzed according to the clinical response to CRT, the 5-year survival rate of patients with CR and IR/SD, or with PD tumors, were 46.0 and 27.3%, respectively (P = 0.02, Figure 2B).

Analysis of the clinical outcome according to Aurora-A levels of the 41 patients who underwent surgery showed that the 2-year or 5-year survival rates of the patients with Aurora-A (+) and Aurora-A (−) tumors were 64.8 and 41.2 or 47.1 and 40.2%, respectively (P = 0.3, Figure 2C). Analysis of the clinical outcome of the same patients according to the histological response to CRT indicated that the 5-year survival rate of patients with grade 2 or 3, or with grade 1 tumors were 61.8 and 21.2%, respectively (P = 0.002, Figure 2D). Furthermore, for better understanding and showing value of Aurora-A, a cox regression analysis was performed. On univariate regression analyses, pathological stage (pStage) and histopathological grade significantly affected postoperative outcome (p = 0.03 and 0.004, respectively), however nuclear and nuclear + cytoplasmic Aurora-A expression did not affect (p = 0.3 and p = 0.4. respectively). On multivariate analysis, pStage and histopathological grade were significant prognostic factors (p = 0.008, hazard ratio = 9.3 and 0.002, hazard ratio = 5.6 respectively), however nuclear and nuclear + cytoplasmic Aurora-A expression were not significant prognostic factors (p = 0.73, hazard ratio = 1.3 and p = 0.91, hazard ratio = 1.2, respectively) (Table 4).

**Table 4:** **Univariate and multivariate analysis of survival in the surgical group of ESCC patients**

Variables (n = 41)	Univariate P	Multivariate P	Hazard ratio	Confidence interval
pStage				
I, II (n = 10)	0.03	0.008	9.3	1.8-48.6
III, IV (n = 31)				1
Histopathological grade				
Grade 2, 3 (n = 21)	0.004	0.002	5.6	1.9-16.6
Grade1 (n = 20)			1	
Nuclear Aurola-A				
(+) (n = 24)	0.30	0.73	1.3	0.3-4.6
(−) (n = 17)			1	
Nuclear + cytoplasmic				
Aurora-A				
(+) (n = 29)	0.40	0.91	1.1	0.2-4.4
(−) (n = 12)			1	

Aurora-A (+)/(−), Aurora-A positive/negative expression.

## Discussion

In the present study, we examined the expression of the Aurora-A protein in ESCC to determine whether such expression might be useful for predicting the response to CRT. Of 78 tumors of ESCC patients, 58.9% of the ESCC tumors were found to have positive expression of Aurora-A, as assessed by a distinct nuclear anti-Aurora-A immunoreaction. This percentage is very similar to that previously reported by Tanaka et al. who reported that 53% of esophageal cancer tissues examined displayed positive nuclear Aurora-A protein expression [11].

When cells undergo various stresses such as the stresses following administration of anticancer drugs and radiation, cell cycle checkpoint and cell cycle arrest mechanisms are activated in order to repair damaged DNA and ensure cell survival. On the other hand, severe DNA damage induces the apoptosis signaling pathway that eliminates unrepaired cells by inducing cell death [18]. Cells exposed to ionizing radiation die via different mechanisms, including apoptosis and mitotic catastrophe [19,20]. Mitotic catastrophe occurs when cells with incompletely replicated genomes or unrepaired DNA damage enter mitosis. Centrosome amplification is an important cause of mitotic catastrophe [21,22]. The present study showed a significant correlation between Aurora-A overexpression (Aurora-A (+) cells), which is correlated to centrosome amplification, and better clinical and histological response to CRT. We consider that overexpression of Aurora-A enables esophageal cancer cells to override the cell cycle check point without repairing DNA damage induced by CRT and enter mitosis, resulting in the occurrence of mitotic catastrophe. There have been reports that describe a correlation between better CRT effect and mitotic catastrophe, which causes dysfunction of G2/M check point regulation, in ESCC patients [7,23]. These results suggest that cell cycle progression without G2 arrest and DNA repair might cause mitotic cell death of ESCC that is related to an effective response to CRT.

In a previous analysis of the correlation of patient survival with Aurora-A levels, the expression of Aurora-A was reported to be associated with poor prognosis of human cancer including esophageal cancer [11]. That result is in contrast to our study in which patients with tumors that overexpressed Aurora-A tended to have a better prognosis in short 2 year period (Figure 2A). Based on this result, we considered that patients whose tumors displayed distinct nuclear overexpression of Aurora-A would have a poor prognosis if they were only treated with surgery without CRT. The combined data suggest that there might be a subgroup of ESCC patients with special characteristics whose malignant potential would be modified by CRT treatment through overriding of the G2/M check point followed by mitotic catastrophe. In the ESCC patients with treated with CRT, lack of Aurora-A expression might be a negative prognostic factor because of poor tumor shrinkage. However when patients have relapse disease in their follow up periods, it might be better prognostic factor. Therefore there was no significant differences on 5 year-survival rates between patients with Aurora-A (+) and Aurora-A (−) tumors. Although further experiments are needed to confirm those phenomena, the present study suggests that favorable responses to CRT can be predicted based on distinct nuclear Aurora-A overexpression in the tumor cells of ESCC patients.

In conclusion, Aurora-A expression in biopsy specimens of primary tumors was associated with a favorable response to CRT of ESCC patients. Assessment of Aurora-A expression in tumor biopsy specimens before therapy, will allow selection of patient response to CRT.

## Conclusions

We demonstrated that CRT was significantly effective in patients who had Aurora-A (+) tumors not only clinically but also histologically. Moreover the patients with Aurora-A (+) tumors tended to have a better prognosis than the patients with Aurora-A (−) tumors. Thus assessment of Aurora-A expression in tumor biopsy specimens before therapy will be useful for selection responders of CRT.

## Abbreviations

CRT: Chemoradiation therapy
ESCC: Esophageal squamous cell carcinoma
CR: Esophageal squamous cell carcinoma
IR/SD: Incomplete response/stable disease of primary lesion
PD: Progressive disease
TNM: Tumor-node-metastasis
